# The association between pubertal timing and quality of life among children and adolescents: a cross-sectional study in Chongqing, China

**DOI:** 10.1265/ehpm.22-00159

**Published:** 2022-12-17

**Authors:** Yang Pu, Yinshuang Tang, Qiuling Shi, Hong Wang

**Affiliations:** 1 School of Public Health, Research Center for Medicine and Social Development, Chongqing Medical University, Chongqing, China; 2 State Key Laboratory of Ultrasound in Medicine and Engineering, Chongqing Medical University, Chongqing, China

**Keywords:** Pubertal timing, Quality of life, Children and adolescents

## Abstract

**Background:**

To determine the relationship between pubertal timing and quality of life (QOL) in children and adolescents and to provide a basis for QOL intervention in pubertal children in the future to promote good adaptation and healthy physical and mental development of children.

**Methods:**

The survey was conducted in one county using a stratified cluster sampling method. The five physiological change items of the Puberty Development Scale (PDS) were used to assess the timing of puberty in students. Compared to students of the same age and the same sex, students who scored higher than the mean + standard deviation (SD) of individual developmental scores were defined as an early pubertal timing group. A 39-item QOL Scale for Children in Puberty was used to assess the QOL of the respondents. Multiple linear regression models were fitted separately for boys and girls.

**Results:**

Of the 7223 students, 3754 (51.97%) were boys and 3469 (48.03%) were girls. The prevalence of early pubertal periods was 16.07%. The total QOL score in the early pubertal timing group (137.16 ± 18.67) was significantly lower than in the normal (on time) group (142.02 ± 17.98) and the late group (142.76 ± 18.35) (F = 37.311, P < 0.001). A multiple linear regression model showed that early pubertal timing was a risk factor for QOL (P < 0.0014), compared with normal and late pubertal timing.

**Conclusions:**

The early pubertal timing was associated with poorer QOL in children and adolescents. More attention should be paid to children with early pubertal timing in intervening children’s QOL during pubertal development. Future longitudinal studies are needed to confirm the association between pubertal timing and QOL.

**Supplementary information:**

The online version contains supplementary material available at https://doi.org/10.1265/ehpm.22-00159.

## Background

Adolescence is a gradual transition from childhood to adulthood, during which individuals undergo dramatic changes in physical, functional, sexual, endocrine, psychological, and behavioral aspects [1, 2]. Adolescence is generally divided into three stages: early, middle, and late adolescence. In early adolescence, also called puberty, children experience a series of events such as growth spurts, breast development, the onset of adrenocortical function, menstruation (in girls), and seminal emission (in boys) [3, 4]. The timing of the first occurrence of these events is referred to as pubertal timing. Pubertal timing is a relative concept describing pubertal development that is early, on time, or late in the context of a reference group or compared to norms [5].

There are subjective and objective methods for assessing pubertal timing. The subjective assessment method mainly uses the PDS to measure the degree of pubertal development, and then calculates the overall or mean value of the scale, using the percentile method or the rank rating method to determine whether the pubertal timing is early, on time, or late [6–8]. The objective assessment method has been used to determine pubertal timing by examining a series of indicators of sexual organ development and secondary sexual characteristics (breast development, pubic hair development, external genitalia development, etc.) [9–12]. The objective assessment method is more accurate in determining the timing of puberty, but it is easily rejected by respondents. Considering the privacy and cooperation of the respondents, as well as the massive human and material resources, the PDS self-assessment scale is considered the most appropriate method for assessing the timing of puberty in a larger population survey [6, 7].

There are many adverse effects of early pubertal timing on children’s physical and mental health. Previous studies have shown that early pubertal timing has been associated with depression, low self-esteem, and other mental health problems [13–15], as well as smoking [16] and alcohol consumption [17, 18]. It also increases the risk of obesity [19], metabolic syndrome, and cardiovascular disease [20, 21] in children and adolescents.

According to the World Health Organization, QOL is determined by physical health, mental state, level of independence, social relationships, environmental factors, and personal beliefs [22]. For children and adolescents, QOL provides a comprehensive assessment of physical health, mental health, and social functioning. Current research on QOL in children and adolescents has focused on some subgroups of children or children with diseases [23–26]. Therefore, studies on the QOL in community and school populations of children and adolescents should also be conducted.

In conclusion, the early pubertal timing has adverse effects on children’s health and development, which in turn may affect QOL. The complex relationship between the timing of puberty and QOL in children and adolescents has not been adequately studied in China. In this study, the PDS was used to assess the timing of puberty in children and adolescents, and the Quality of Life Scale for Children in Puberty [27, 28] was used to assess QOL, investigate differences in QOL at different times of puberty, and provide a basis for future interventions on the QOL of life of pubertal children to promote good adaptation and healthy physical and mental development.

## Methods

### Study design and participants

A multistage cluster sampling method was used to select 11 schools in the QiJiang district, Chongqing, China. First, the QiJiang district, Chongqing, was selected as the survey site. Second, five middle schools and six primary schools were selected from this district. Finally, 3 to 8 classes from each grade from the third to sixth grade of primary schools and seventh to ninth grade of middle schools were selected, and all students in the classes studied were included as participants. When investigating, each class had one or two investigators. Before the start of the survey, the investigator explained the purpose and content of the survey and the precautions for filling in the questionnaire. We also explained the concept of puberty to the third grade students. Although third grade children are outside the pubertal period, the correct knowledge and attitude toward puberty will help children to pass through this stage more smoothly. Guideline for Health Education in Primary and Secondary Schools, issued by Ministry of Education of the People’s Republic of China in 2008, mentioned that three to four grade students should learn about growth and adolescent health, including the human life cycle such as birth and development, and the functions of the main organs of the body of children and adolescents [29]. Moreover, in the process of filling out the questionnaire, the investigator explained the questions encountered by the students one-on-one. Those who were unwilling to participate in the survey and did not understand the content of the study were excluded. This survey was conducted in December 2017. A total of 7446 students were surveyed by questionnaire, and 7223 valid participants were finally included in this analysis.

### Measurements

#### Demographic characteristics

The following information was collected from the students using a self-administered questionnaire: age, gender (boy/girl), without siblings (yes/no), boarding school student (yes/no), left-behind children (yes/no), family financial situation (good/moderate/poor), physical condition (good/moderate/bad), self-reported physique (low weight, normal weight, overweight), and academic performance (good/moderate/bad). In China, left behind children usually refer to minors under the age of 16 whose parents are working outside the home or one of them is working outside the home and the other has no guardianship ability.

#### Pubertal timing

The timing was measured using the PDS with five items [6–8]. The PDS for boys included items related to changes in growth spurt, body hair growth, facial hair growth, deepening of the voice, and skin (especially pimples). The PDS for girls included items related to body hair growth, growth spurt, and skin changes (especially pimples), age at menarche, and breast growth. The scale for scoring development contained the following options: ‘not yet started’ (1 point), ‘barely started’ (2 points), ‘definitely started’ (3 points), or ‘seems completed’ (4 points). The options for the menarche item were yes (4 points) and no (1 point). The pubertal timing was evaluated using the total PDS score divided by the number of items. We defined three groups for pubertal timing: early (>mean + 1 SD for peers of the same age and sex), on time (within mean ± SD), and late (<mean − 1 SD) [16, 30].

#### QOL

In this study, the QOL Scale for children in puberty was used. All elements were derived mainly from the Inventory of Subjective Life Quality for Children and Adolescents (ISLQ) [31], CAQOL [32], and the Chinese version of Peds QL4.0 [33]. Furthermore, we consider the basic characteristics of adolescent children. A scale with 39 elements and four subscales (physiological (8 items), mental (11 items), social (14 items), and pubertal well-being (6 items)) was formed (see Additional file 1). There were two types of items: the frequency of a certain phenomenon (type 1) and the satisfaction of participants with their life situation (type 2). The recall period was the last three months. Items were rated on a 5-point Likert scale (type1: 5 = never, 1 = always; type2: 5 = very satisfied, 1 = very dissatisfied), with the values of item 23 reversed (5 = always, 1 = never). The total scale score indicated the participant’s QOL status, with higher total scores indicating a better QOL. The reliability, validity, and acceptability of this scale have been confirmed among primary and middle school students (aged 9 to 15) in Chongqing [27, 28].

### Statistical analysis

Statistical analysis was performed using SPSS (IBM SPSS 22.0, SPSS Inc). Demographic characteristics of the different genders were analyzed using the chi-square test or the t test. The QOL score between groups with different characteristics was assessed with an F or t test. Multiple linear regression models were fitted separately for boys and girls. In the total model, the QOL score and the scores of the four subscales were used as dependent variables, demographic characteristics as covariates, and the timing of puberty as independent variables. The trichotomous variables were transformed into dummy variables, and the focus category of concern was used as a reference. Because we performed four hypothesis tests for nine factors on the same data, resulting in an increased probability of Type I errors, the Bonferroni method was used to correct α. Therefore, for linear regression, the α was approximately 0.0014 (0.05/36), and for other analyses, the α was 0.05. All analyses were performed using two-sided tests.

## Results

### Characteristics of the sample

A total of 3754 (51.97%) participants were boys and 3469 (48.03%) were girls. The average age of the boys and girls was 11.49 ± 2.01 and 11.50 ± 2.05 years, respectively. Of the boys, 53.60% were left-behind children, and of the girls, 53.56% were left-behind children. Boys reported a higher rate of good physical condition than girls (79.41% vs 74.46%, *χ*^2^ = 30.747, *P* < 0.001). Regarding the self-reported physique, the rate of thin physique in boys was higher than in girls (32.05% vs 22.40%, *χ*^2^ = 99.459, *P* < 0.001). Girls showed a poorer academic performance than boys (19.49% vs 26.87%, *χ*^2^ = 55.811, *P* < 0.001). (Table 1).

**Table 1:** Descriptive statistics for the participants (N = 7223)

**Variable**	**Boys (n = 3754)**		**Girls (n = 3469)**		***χ*^2^/t**	***P***

	**N (mean)**	**% (SD)**	**N (mean)**	**% (SD)**		
Age	11.49	2.01	11.50	2.05	−0.233	0.816
Without siblings						
Yes	777	20.70	548	15.80	28.909	<0.001
No	2977	79.30	2921	84.20		
Boarder						
Yes	918	24.45	908	26.17	2.826	0.093
No	2836	75.55	2561	73.83		
Left-behind children						
Yes	2012	53.60	1859	53.59	0.000	0.995
No	1742	46.40	1610	46.41		
Family financial situation						
Good	1379	36.73	1225	35.31	11.935	0.003
Moderate	1819	48.45	1806	52.06		
Poor	556	14.81	438	12.63		
Students’ physical condition						
Good	2981	79.41	2583	74.46	30.747	<0.001
Moderate	588	15.66	717	20.67		
Bad	185	4.93	169	4.87		
Self-reported physique						
Thin	1203	32.05	777	22.40	99.459	<0.001
Normal	1916	51.04	1893	54.57		
Overweight	635	16.91	799	23.03		
Academic performance						
Good	1051	28.00	1038	29.92	55.811	<0.001
Moderate	1694	45.13	1755	50.59		
Bad	1009	26.87	676	19.49		

### The pubertal timing distribution classified by PDS score

Table 2 shows the mean, SD, mean minus SD, and mean plus SD of the PDS score for different genders and age groups. The percentage of participants at each pubertal stage by gender and age groups was displayed in Additional file 2. The prevalence of children in early puberty was 16.07%. Among boys, 660 (17.58%) were in the early puberty stage, 2538 (67.61%) were normal (on time) and 556 (14.81%) were late. Among female students, 501 (14.44%) were early pubertal timing, 2575 (74.23%) were normal pubertal timing, and 393 (11.33%) were late pubertal timing. (Table 2 and Additional file 2)

**Table 2:** Mean and SD of PDS score for boys and girls aged 8∼15 years

**Age group**	**Boys**					**Girls**				

	**N**	**Mean**	**SD**	**Mean − SD**	**Mean + SD**	**N**	**Mean**	**SD**	**Mean − SD**	**Mean + SD**
8∼	228	1.58	0.63	0.95	2.22	230	1.37	0.43	0.94	1.81
9∼	534	1.55	0.53	1.02	2.08	515	1.42	0.44	0.98	1.86
10∼	586	1.43	0.46	0.97	1.89	501	1.49	0.48	1.01	1.98
11∼	528	1.46	0.45	1.01	1.91	492	1.78	0.59	1.19	2.37
12∼	586	1.65	0.50	1.15	2.16	465	2.18	0.62	1.57	2.80
13∼	528	2.01	0.54	1.47	2.56	514	2.50	0.51	2.00	3.01
14∼	507	2.23	0.55	1.69	2.78	529	2.64	0.47	2.17	3.10
15∼	257	2.42	0.48	1.94	2.90	223	2.77	0.45	2.32	3.22
Total	3754	1.75	0.61	1.14	2.36	3469	2.01	0.72	1.29	2.73

### QOL scores between different characteristics in children and adolescents

Table 3 shows that the timing of puberty, gender, boarding school status, family financial situation, student physical condition, self-reported physique, and academic performance had a significant association with QOL (all *P* < 0.05). The mean QOL score in the early pubertal timing group (137.16 ± 18.67) was significantly lower than in the normal group (142.02 ± 17.98) and the late group (142.76 ± 18.35) (F = 37.311, *P* < 0.001). The QOL scores of boys were slightly higher than those of girls (t = 3.361, *P* = 0.040). Students with a good financial situation, good physical condition, self-reported normal physique, and good academic performance had higher QOL scores. (Table 3)

**Table 3:** Comparison of QOL between different characteristics in children and adolescents

**Variable**		**Total scores** **(Mean ± SD)**	**F/t**	***P***
Pubertal timing	Early	137.16 ± 18.67	37.311	<0.001
	On time	142.02 ± 17.98		
	Late	142.76 ± 18.35		
Gender	Boys	141.76 ± 18.54	3.361	0.040
	Girls	140.88 ± 17.89		
Without siblings	Yes	141.48 ± 18.32	0.308	0.758
	No	141.30 ± 18.22		
Boarder	Yes	137.85 ± 16.90	−9.501	<0.001
	No	142.51 ± 18.52		
Left-behind students	Yes	140.77 ± 17.99	−2.834	0.005
	No	141.99 ± 18.50		
Family financial situation	Good	145.43 ± 19.11	127.867	<0.001
	Moderate	139.94 ± 17.02		
	Poor	135.72 ± 17.92		
Students’ physical condition	Good	143.72 ± 17.78	228.800	<0.001
	Moderate	134.20 ± 17.10		
	Bad	129.81 ± 18.39		
Self-reported physique	Thin	140.40 ± 18.44	70.629	<0.001
	Normal	143.45 ± 17.99		
	Overweight	137.02 ± 17.72		
Academic performance	Good	145.94 ± 18.05	175.803	<0.001
	Moderate	141.61 ± 17.80		
	Bad	135.05 ± 17.52		

### Multiple linear regression analysis between pubertal timing and QOL (total and subscale) scores for boys and girls

As shown by the unadjusted linear regression model, the QOL scores were lower in both boys and girls in the early pubertal timing group than in the normal group (b_boy_ = 4.615, b_girl_ = 5.304, P < 0.0014) and in the late group (b_boy_ = 5.915, b_girl_ = 5.092, P < 0.0014). After adjusting for covariates, the associations between the early pubertal timing group and the normal group (b_boy_ = 3.889, b_girl_ = 3.991, P < 0.0014) and the late group (b_boy_ = 5.822, b_girl_ = 4.244, P < 0.0014) remained significant.

Regarding the characteristics, the QOL scores were significantly higher among students with a good family financial situation (b_boy_ = 4.082, b_girl_ = 4.906, P < 0.0014), good physical condition (b_boy_ = 11.603, b_girl_ = 10.734, P < 0.0014), normal physique (b_boy_ = 3.646, b_girl_ = 4.887, P < 0.0014), good (b_boy_ = 7.007, b_girl_ = 8.234, P < 0.0014) and moderate (b_boy_ = 4.936, b_girl_ = 4.730, P < 0.0014) academic performance in boys and girls. Furthermore, older age was associated with lower QOL scores in girls (b = −1.069, P < 0.0014). (Table 4)

**Table 4:** Multiple linear regression analysis between pubertal timing and QOL scores for different genders

**Parameter**	**Boys**					**Girls**				

	**Physiological**	**Mental**	**Social**	**Pubertal**	**Total scores**	**Physiological**	**Mental**	**Social**	**Pubertal**	**Total scores**
Unadjusted										
Normal pubertal timing vs. early pubertal timing	1.541*	0.877	2.010*	0.187	4.615*	1.374*	1.434*	2.361*	0.135	5.304*
Late pubertal timing vs. early pubertal timing	2.251*	1.850*	1.938*	−0.123	5.915*	1.798*	1.364*	2.554*	−0.623	5.092*
Adjusted										
Normal pubertal timing vs. early pubertal timing	1.314*	0.722	1.645*	0.207	3.889*	1.120*	1.133*	1.716*	0.021	3.991*
Late pubertal timing vs. early pubertal timing	2.268*	1.876*	1.854*	−0.175	5.822*	1.780*	1.274	2.102*	−0.913*	4.244*
Age	−0.224*	−0.048	0.102	0.248*	0.079	−0.621*	−0.555*	−0.339*	0.446*	−1.069*
Without siblings vs. with siblings	0.279	0.093	−0.487	−0.028	−0.143	−0.031	−0.162	−0.617	−0.067	−0.877
Not boarder vs. boarder	0.332	0.124	0.376	−0.355	0.478	0.418	0.292	0.913	−0.508	1.115
Not left-behind children vs left-behind children	0.152	−0.427	0.106	0.077	−0.092	0.146	−0.060	0.685	0.188	0.959
Good family financial situation vs. bad family financial situation	0.499	0.631	2.499*	0.453	4.082*	0.763	0.412	3.206*	0.525	4.906*
Moderate family financial situation vs. bad family financial situation	−0.107	0.249	1.337	0.197	1.677	0.245	−0.054	2.189*	0.154	2.534
Good physical condition vs. bad physical condition	2.642*	2.485*	5.388*	1.088*	11.603*	3.427*	2.500*	3.754*	1.052*	10.734*
Moderate physical condition vs. bad physical condition	0.667	1.074	1.974	0.505	4.220	1.509*	0.862	0.828	0.259	3.457
Thin vs. normal physique	−0.582	−0.779	−1.051*	−0.197	−2.610*	−0.332	−0.510	−0.605	−0.243	−1.689
Overweight vs. normal physique	−0.466	−0.643	−2.089*	−0.448	−3.646*	−0.710*	−1.224*	−2.384*	−0.569*	−4.887*
Good academic performance vs. bad academic performance	0.590	1.609*	3.719*	1.090*	7.007*	0.362	1.586*	5.107*	1.179*	8.234*
Moderate academic performance vs. bad academic performance	0.458	1.201*	2.735*	0.542*	4.936*	0.089	0.554	3.205*	0.882*	4.730*

The numbers in the table represent the value of b.

*P < 0.0014

Compared to boys with an early pubertal timing, students in the normal group had significantly higher scores in the physiological dimension (b = 1.314, P < 0.0014) and the social dimension (b = 1.645, P < 0.0014). Students in the late group had significantly higher scores in the physiological dimension (b = 2.268, P < 0.0014), the mental dimension (b = 1.876, P < 0.0014), and the social dimension (b = 1.854, P < 0.0014) in boys. Among the girls, the students in the normal pubertal timing group had significantly higher scores in the physiological dimension (b = 1.120, P < 0.0014), mental dimension (b = 1.133, P < 0.0014), and social dimension (b = 1.716, P < 0.0014); students in the late group also had significantly higher scores in the physiological dimension (b = 1.780, P < 0.0014) and social dimension (b = 2.102, P < 0.0014) and had significantly lower scores in the pubertal dimension (b = −0.913, P < 0.0014). Additionally, students with good physical condition had significantly higher QOL scores in the total scale and in the specific domain subscales than students with poor physical condition. (Table 4)

## Discussion

Our results showed that the early pubertal timing was associated with poorer QOL in children and adolescents, which is similar to the results of the study of Japanese students by Fujimura et al. [34]. Compared to previous studies [16, 18, 34], our study made some advances: First, this study used the PDS and the QOL scale for pubescent children to further explore the relationship between pubertal timing and the dimensions of QOL. Furthermore, our survey was conducted among students in grades 3 through 9 (8 to 15 years old), covering almost all age groups in early and middle adolescence.

The results showed that the differences in QOL scores between the group with an early puberty timing and the group without an early puberty timing were found mainly in physiological, mental and social dimensions in both boys and girls. The physiological dimension includes two factors: somatization symptoms and sleep status. The mental dimension includes negative and aggressive emotions. Each item in these factors is assigned a score based on the frequency of occurrence. The lower the frequency of occurrence, the higher the QOL in the physiological and psychological dimensions. Items in the social dimension are scored based on satisfaction, with higher scores indicating better quality of life. The social dimension includes family life, school life, peer relationships and appearance experience factors. The results indicated that children with early pubertal timing were less satisfied with family life, school life, peer relationships and appearance experience than children without early pubertal timing.

The early pubertal timing has been shown to be associated with internalizing symptoms [35, 36] and shorter duration of sleep and later bedtimes [8]. Additionally, the early pubertal timing is considered a risk factor for developing aggressive behavior [37, 38], depression, and anxiety [39, 40]. Due to the many problems that early onset of puberty causes for children in physical, mental and social aspects, the QOL of children with early onset of puberty is worse in physical, mental and social aspects. Furthermore, for girls, the pubertal dimensions scores were significantly higher in the early pubertal timing group than in the late group. Possibly, because early menstruation and other physiological changes in girls in the early puberty group attracted the attention of themselves and their parents, they paid more attention to the relevant content of puberty and learned about adolescence earlier.

According to the PDS score, the rate of early pubertal timing among children and adolescents aged 8–15 years in this study was 16.07% (1161/7223), which was lower than that in students aged 11–16 years in Hunan Province (19.1%) [18] but close to the rates in Taiwan (16.6%) [41] and Chongqing (16.4%) [42]. This might be due to the difference in the study subject. The Hunan study population consisted of junior high school students rather than elementary school students, who had already developed to different degrees; therefore, the rate of early pubertal timing was different. Additionally, our study used the rank scoring method. For boys aged 8 or 10 years and girls aged 8 or 9 years, the mean minus SD value was less than 1, while the PDS values of all students were ≥1, which meant that no students were included in the group with late pubertal timing. This suggests that there are numerous children under 10 years of age for whom physical development has not yet begun, and that PDS may not be able to distinguish children with delayed development or that there is no late pubertal timing in these children. However, this had little effect on the identification of children with early pubertal timing.

Additionally, we found that physical condition and academic performance also have a significant association with the QOL of children and adolescents. Children with better academic performance receive more attention and care from parents, teachers, and classmates. They have a better sense of life at home, at school, and among their peers. Furthermore, students with better academic performance may have a stronger ability to learn and acquire correct knowledge, which is conducive to the formation of correct cognition in these children. Children with poor physical health, who suffer from symptoms of illness or discomfort, naturally have poor QOL. When studying the relationship between QOL and factors of interest, these confounding factors (such as academic performance) need to be taken into account in order to draw more accurate conclusions.

In interpreting our results, some caveats should be noted. First, we adjusted for α in the linear regression analysis, which may have resulted in us missing some meaningful results. Second, this study used at subjective assessment method (the PDS) to determine pubertal timing, which can differ from an objective assessment method (assessment by professional examiners according to the Tanner staging criteria). Thirdly, the reliability, validity, and acceptability of QOL Scale for children in puberty hadn’t been confirmed for 8-year-old children. As for this present study, the measurement has shown good reliability in 8-year-old children. The Cronbach’s alpha of the total scale was 0.877, and 0.781, 0.773, 0.841 and 0.614 for each subscale, respectively. During the survey, we found that the major of 8-year-old children could complete the scale with high quality. It indicates that the scale is acceptable for 8-year-old students. The validity of this scale in 8-year-old children need to be verified in the future. Finally, the study was a cross-sectional study and causality cannot be established with this study design.

## Conclusions

The early pubertal timing was associated with poorer QOL in children and adolescents. Future longitudinal studies are needed to confirm the association between pubertal timing and QOL. Measures should be taken to improve the QOL of children with early pubertal timing to promote good adaptation and healthy physical and mental development of children.

## Abbreviations

QOL: quality of life
PDS: Puberty Development Scale
SD: Standard deviation

## Supplementary information

**Additional file 1:** The Quality of Life Scale for children in puberty.

**Additional file 2:** Pubertal timing in different age groups and genders.

## Declarations

### Availability of data and materials

The datasets analyzed during the current study are available from the corresponding author on reasonable request.
