# Supplementary material for: The association between pubertal timing and quality of life among children and adolescents: a cross-sectional study in Chongqing, China
Source: Environ Health Prev Med. 2022 Dec 17;27:49. doi: 10.1265/ehpm.22-00159 (PMC9792678; doi:10.1265/ehpm.22-00159)
Supplement: Supplementary file 1 — Additional file 1: The Quality of Life Scale for children in puberty. [file ehpm-27-049-s001.docx]

**the QOL Scale for children in puberty**

Please recall the frequency of the following situations and your feelings in the last three months, and tick the appropriate box according to your actual situation.

|  | Never | Almost | Sometimes | Often | Always |
| --- | --- | --- | --- | --- | --- |
| 1.Did you have trouble falling asleep? |  |  |  |  |  |
| 2.Did you feel tired when you wake up in the morning? |  |  |  |  |  |
| 3.Did you have a poor mental state during the day and can't lift your spirits? |  |  |  |  |  |
| 4.Did you feel tightness in your chest? |  |  |  |  |  |
| 1. Did you feel stomach pain? |  |  |  |  |  |
| 6.Did you feel dizzy? |  |  |  |  |  |
| 7.Did you feel weak and powerless? |  |  |  |  |  |
| 8.Did you feel numbness or tingling in your hands and feet? |  |  |  |  |  |
| 9.Did you have no interest in anything? |  |  |  |  |  |
| 10.Did you have no confidence in yourself? |  |  |  |  |  |
| 11.Did you worry that you are not as good as other students? |  |  |  |  |  |
| 12.Did you regret what you have done? |  |  |  |  |  |
| 13.Did you worry that you can't do things well? |  |  |  |  |  |
| 14.Did you worry about whether others really like to play with you? |  |  |  |  |  |
| 15.Did you get nervous and anxious easily? |  |  |  |  |  |
| 16.Did you have an uncontrollable urge to hit someone? |  |  |  |  |  |
| 17.Did you feel that someone is laughing at you behind your back? |  |  |  |  |  |
| 18.Did you ever threaten people you know? |  |  |  |  |  |
| 19.Did you have a hard time controlling your temper? |  |  |  |  |  |
| 20.Did you never consider the feelings of others in doing things? |  |  |  |  |  |
| 21.Were you discriminated against or excluded by other students? |  |  |  |  |  |
| 22.Did you have difficulty getting along with your classmates? |  |  |  |  |  |
| 23.Did you actively want to learn about puberty? |  |  |  |  |  |
| 24.Did you get scared when you think about puberty? |  |  |  |  |  |
| 1. Does the thought of puberty bother you? |  |  |  |  |  |
|  | Very dissatisfied | Dissatisfied | Neither satisfied nor dissatisfied | Satisfied | Very satisfied |
| 26.Were you satisfied with your appearance? |  |  |  |  |  |
| 27.Were you satisfied with your body type? |  |  |  |  |  |
| 28.Were you satisfied with your relationship with your parents? |  |  |  |  |  |
| 29.Were you satisfied with your position in the family? |  |  |  |  |  |
| 30.Were you satisfied with your living environment? |  |  |  |  |  |
| 31.Were you satisfied with your relationship with your classmates around you? |  |  |  |  |  |
| 32.Were you satisfied with the state of your classes? |  |  |  |  |  |
| 33.Were you satisfied with your motivation to participate in school activities? |  |  |  |  |  |
| 34.Were you satisfied with your initiative in studying? |  |  |  |  |  |
| 35.Were you satisfied with your school life? |  |  |  |  |  |
| 36.Were you satisfied with your ability to learn and accept new things? |  |  |  |  |  |
| 37.Were you satisfied with the knowledge you have acquired about puberty? |  |  |  |  |  |
| 38.Were you satisfied with the ways of acquiring knowledge about puberty? |  |  |  |  |  |
| 39.Were you satisfied with the way you get along with your classmates of the opposite sex? |  |  |  |  |  |
