# Supplementary material for: The association between pubertal timing and quality of life among children and adolescents: a cross-sectional study in Chongqing, China
Source: Environ Health Prev Med. 2022 Dec 17;27:49. doi: 10.1265/ehpm.22-00159 (PMC9792678; doi:10.1265/ehpm.22-00159)
Supplement: Supplementary file 2 — Additional file 2: Pubertal timing in different age groups and genders. [file ehpm-27-049-s002.docx]

**Additional file 2**

Pubertal timing in different age groups and genders

| Gender | Age | Early | % | On time | % | Late | % |
| --- | --- | --- | --- | --- | --- | --- | --- |
| Boys | 8~ | 34 | 14.91 | 194 | 85.09 | 0 | 0.00 |
|  | 9~ | 81 | 15.17 | 342 | 64.04 | 111 | 20.79 |
|  | 10~ | 84 | 14.33 | 502 | 85.67 | 0 | 0.00 |
|  | 11~ | 86 | 16.29 | 302 | 57.20 | 140 | 26.52 |
|  | 12~ | 119 | 20.31 | 382 | 65.19 | 85 | 14.51 |
|  | 13~ | 103 | 19.51 | 325 | 61.55 | 100 | 18.94 |
|  | 14~ | 107 | 21.10 | 315 | 62.13 | 85 | 16.77 |
|  | 15~ | 46 | 17.90 | 176 | 68.48 | 35 | 13.62 |
|  | Total | 660 | 17.58 | 2538 | 67.61 | 556 | 14.81 |
| Girls | 8~ | 26 | 11.30 | 204 | 88.70 | 0 | 0.00 |
|  | 9~ | 63 | 12.23 | 452 | 87.77 | 0 | 0.00 |
|  | 10~ | 84 | 16.77 | 299 | 59.68 | 118 | 23.55 |
|  | 11~ | 106 | 21.54 | 325 | 66.06 | 61 | 12.40 |
|  | 12~ | 53 | 11.40 | 337 | 72.47 | 75 | 16.13 |
|  | 13~ | 53 | 10.31 | 402 | 78.21 | 59 | 11.48 |
|  | 14~ | 86 | 16.26 | 387 | 73.16 | 56 | 10.59 |
|  | 15~ | 30 | 13.45 | 169 | 75.78 | 24 | 10.76 |
|  | Total | 501 | 14.44 | 2575 | 74.23 | 393 | 11.33 |
| Total |  | 1161 | 16.07 | 5113 | 70.79 | 949 | 13.14 |
